# Supplementary material for: Vibrational fingerprint of localized excitons in a two-dimensional metal-organic crystal
Source: Nat Commun. 2018 Nov 8;9:4703. doi: 10.1038/s41467-018-07190-1 (PMC6224418; doi:10.1038/s41467-018-07190-1)
Supplement: Supplementary file 1 — Supplementary Information [file 41467_2018_7190_MOESM1_ESM.pdf]

Supplementary Information

Vibrational fingerprint of localized excitons in a 2D metalorganic crystal

M. Corva,[1,2] A. Ferrari,[1,†] M. Rinaldi,[1] Z. Feng,[1,‡] M. Roiaz,[3] C. Rameshan,[3] G. Rupprechter,[3] R. Costantini,[1,2] M. Dell'Angela,[2] G. Pastore,[1] G. Comelli,[1,2] N. Seriani,[4] and E. Vesselli[1,2,*]

[1]Physics Department, Università degli Studi di Trieste, via A. Valerio 2, Trieste 34127, Italy.

[2]Istituto Officina dei Materiali CNR-IOM, S.S. 14 km 163.5, Area Science Park, Basovizza, Trieste 34149, Italy.

[3]Institute of Materials Chemistry, Vienna University of Technology, Getreidemarkt 9-BC-01, Vienna A-1060, Austria.

[4]The Abdus Salam International Centre for Theoretical Physics, Str. Costiera 11, Trieste 34151, Italy.

*Correspondence to: evesselli@units.it

†Current address: ICAMS, Ruhr-Universität Bochum, Universitätsstr. 150, Bochum 44801, Germany.

‡Current address: Department of Chemical Engineering and Materials Science, University of California, Irvine, Irvine, California 92697, United States.

Supplementary Note 1

An extensive set of *ab initio* calculations was performed to investigate the possible effects involved in the generation of the vibronic multiplet observed in the IR-Vis SFG spectra. Here in the following we report a list of the obtained results, which led to the conclusions addressed in the manuscript.

Cooperative adsorption hypothesis. The first hypothesis that we investigated consisted in assuming that the anomalous multiplicity was due to the cooperative adsorption of multiple CO molecules on a single FePc. The chemisorption of a first CO molecule would thus rearrange the structure of the system in such a way that further CO adsorption would become energetically favorable. This is the cooperative adsorption hypothesis. The initial simulation system is composed of an isolated FePc molecule only. This is justified as, experimentally, the FePc molecules are decoupled from the Ir(111) surface by the graphene sheet and, since we have a π-π bonding, the graphene layer is not expected to affect drastically the properties of the phthalocyanine.[1,2] The simulations show that the total magnetization is equal to 2.04 μB, thus the system is in a triplet state. Moreover, the central iron atom has formally a +2 charge. To probe the multiple carbonylation hypothesis, up to three CO molecules have been coadsorbed on FePc. The carbonylation of the FePc quenches the spin of the system, thus the magnetization becomes equal to 0.00 μB, i.e. the system is in a singlet state. Calculations show that the only stable adsorption geometry is that reported in Fig. 2A, while multiple adsorption is energetically unfavorable. The calculated adsorption energy for the single CO, -0.34 eV, is in good agreement with the experimental value of -0.3±0.1 eV. For di-carbonylation, the adsorption energy is +0.23 eV/CO, and for tri-coordination +0.22 eV/CO thus ruling out cooperative mechanisms.

***Ab initio* thermodynamics.** When describing finite, constant pressure and temperature conditions, the relevant quantity is the total Gibbs free energy G^*, rather than the adsorption energy.[3] The former is obtained from DFT total energies. For the adsorption of a single CO molecule at each metal center, the relevant quantity is the difference:

$$G^*(T,p) = G(T,p) - \mu_{FePc}(T,p) - \mu_{CO}(T,p) \quad (1)$$

where G is the Gibbs free energy of the adsorbed system, and μ_{FePc} and μ_{CO} are the chemical potentials of the FePc and CO molecules, respectively. The DFT total energies are evaluated within a given volume V of the unit cell and therefore the Gibbs free energy can be expressed in terms of the Helmholtz free energy plus the volume contributions, i.e.:

$$G(T,p) = F(T,V) + pV \quad (2)$$

The term pV can be neglected since p is of the order of 10 mbar in the present case, and V is smaller than 10^4 Å^3, yielding $pV < 6\ 10^{-5}$ eV, much lower than the energy differences characterizing nonequivalent configurations (0.1 eV). The Helmholtz free energy can be further detailed as:

$$F(T,V) = E + F_{vibr}(T,V) \quad (3)$$

with

$$F_{vibr}(T,V) = E_{vibr}(T,V) - T \cdot S_{vibr}(T,V) \tag{4}$$

that includes energy and entropy contributions from the vibrational modes of the system. However, since vibrational energies F_{vibr} are generally small with respect to adsorption energies, they can be neglected as a first approximation.

Using all the assumptions above we obtain that

$$G^*(T,p) \cong E - \mu_{FePc} - \mu_{CO}(T,p) \tag{5}$$

The chemical potential for CO can be adequately parametrized with the expression valid for ideal gases where

$$\mu_{CO}(T,p) = E_{CO} + \mu_{CO}(T,p_{atm}) + k_B T \cdot ln\left(\frac{p}{p_{atm}}\right) \tag{6}$$

From thermochemical tables μ_{CO}(300 K, p_{atm}) = -0.53 eV. Collecting all the terms and setting the zero of the free energy scale for the clean FePc molecule (no CO adsorbed), the free energy difference that contributes in the CO adsorption process is

$$\Delta G^*(T,p) \cong E_{ads} - \mu_{CO}(T,p_{atm}) - k_B T \cdot ln\left(\frac{p}{p_{atm}}\right) \tag{7}$$

In the isothermal-isobaric ensemble, the probability of CO adsorption on a FePc molecule is

$$P = \frac{e^{-\beta \Delta G^*(T,p)}}{1 + e^{-\beta \Delta G^*(T,p)}} \tag{8}$$

with $\beta = 1/(k_B T)$. Consequently, the coverage of CO at the Fe sites of the FePc monolayer is

$$\frac{\theta}{\theta_{sat}} = \frac{r(T,p)e^{-\beta \Delta G^*(T,p)}}{1 + r(T,p)e^{-\beta \Delta G^*(T,p)}} \tag{9}$$

where r(T,p) is the ratio between the number of available molecules in the gas phase N_{gas}(p) and the number of available adsorption sites N_{sites}:

$$r(T,p) = \frac{N_{gas}(T,p)}{N_{sites}} = \frac{p \cdot V_{chamber}}{k_B T} \cdot \frac{A_{unit\ cell}}{A_{sample}} \tag{10}$$

In our setup the volume of the high pressure cell is $V_{chamber} \sim 10^{-3}$ m^3, the surface area of the unit cell of the FePc monolayer is $A_{unit\ cell} \sim 200$ Å^2 and the sample surface is $A_{sample} \sim 50$ mm^2. The full *ab initio* thermodynamic expression for the surface coverage is finally

$$\frac{\theta}{\theta_{sat}} = \frac{p \cdot B(T,p)}{1 + p \cdot B(T,p)}$$
$$B(T,p) = r(T,p) \cdot \frac{e^{\beta[\mu_{CO}(T,p_{atm}) - E_{ads}]}}{p_{atm}} \tag{11}$$

which is formally equivalent to the well-known Langmuir formula for isothermal adsorption. The curve plotted in Fig. 3 together with the experimental data points is directly obtained from this *ab initio* model, with no fitted parameters. This plot shows that the experimental data are well reproduced by the adsorption of only one CO molecule per iron phthalocyanine. Multiple carbonylation of FePc remains thermodynamically unfavorable, even when taking into account the effect of temperature and pressure. Further calculations of the adsorption energy as a function of the distance of the carbon atom of CO from the equilibrium adsorption length show no barrier for the single adsorption process, which is therefore not activated. The conclusions again confirm the exclusion of the cooperative adsorption hypothesis.

The dipole-dipole interaction hypothesis. We have shown that the relative intensities of the multiple peaks observed in the SFG spectra depend on the degree of order of the FePc monolayer (see main text). Therefore, interaction between neighboring phthalocyanines plays a role. The dipole-dipole interaction hypothesis is based on a direct interaction of the dipoles of CO molecules adsorbed at adjacent phthalocyanines. We considered the potential energy for this interaction: $U(r) = p^2_{CO}/(4\pi\varepsilon_0 r^3)$. Setting r = 13.6 Å and p_{CO} = 0.112 D,[4] we obtain $U = 3 \times 10^{-6}$ eV, three orders of magnitude smaller than the peak spacing in the SFG spectra. Thus, the dipole-dipole interaction hypothesis had to be discarded.

The harmonic coupling hypothesis. Another possible hypothesis about the nature of the interaction between the (CO)FePc molecules consists in assuming that the CO-CO interaction is indirectly mediated by the vibrations of the FePc 2D molecular lattice. We have calculated the frequency of the C-O stretching mode for this system by using density functional perturbation theory (DFPT). A finite dispersion of phonon modes in the first Brillouin zone would have demonstrated that intermolecular coupling is responsible for the appearance of several peaks in the SFG spectra. Instead, at different k-points the modes differ at most by 3 cm^{-1}, i.e. no direct effect on the vibrational modes could be observed, leading to the rejection of the harmonic coupling hypothesis, consistently with the PM-IRAS measurements.

The excitonic hypothesis. After discarding the harmonic coupling hypothesis, where the interaction is of pure phononic nature, we analyze the influence of electronic effects. The excitonic hypothesis assumes that, upon illumination with visible light in a SFG experiment, a fraction of the molecules turns in an electronically excited state. Provided the lifetime of the latter is long enough, the SFG process occurs on the set of populated states. The multiplet of peaks would then originate from the non-equivalent vibrational modes of both the electronically excited and ground states. To prove the excitonic hypothesis, the vibrational modes of an excited state have been investigated. Since the ground state is a singlet, a virtually excited state was obtained by imposing a total magnetization of the system equal to 2.00 μB in the calculations. The vibrational frequencies were calculated both with DFT and with DFT+U. DFT yields frequencies of 1994, and 1996 cm^{-1} for the singlet and the lowest-lying triplet states, respectively. The obtained triplet state is associated with the HOMO to LUMO transition. DFT+U yields values of 2016 and 2049 cm^{-1},

respectively, and 2055 cm^{-1} for another excited configuration. As shown in Supplementary Figure 2, imposing a total magnetization of 2.00 μB in the calculation forces one electron originally occupying a HOMO-1 state to be lifted into a LUMO+1 state. The other excited state was instead obtained by imposing a fixed occupation on the orbitals, forcing the second highest minority spin state below the Fermi energy to be empty and the second lowest majority-spin state above the Fermi energy to be occupied. The vibrational energy differences are compatible with the experimentally observed values, thus lending support for the excitonic hypothesis.

Dependence of the IR-Vis SFG spectra on temperature and laser parameters. In Supplementary Figure 3 we plot the IR-Vis SFG spectra collected *in situ* in the C-O stretching region on the FePc monlayer on graphene in 6 (left) and 12 (right) mbar CO for increasing surface temperature, starting from 300 K (top). The relative amplitude of the features is plotted in Supplementary Figure 4 (left axis) together with the inhomogeneity gaussian broadening (right axis). As a rule of thumb, the lowest energy features gain relative intensity with annealing temperature at the expense of the peak at 2011 cm^{-1}. This is accompained with a progressive broadening of the resonances due to an increased gaussian width, starting from 320 K. By plotting the same quantities measured at room temperature but on samples obtained with a different 2D crystal growth temperature (Supplementary Figure 5), a similar trend can be observed. In the latter case the features at 1986 and 1992 cm^{-1} increase their relative amplitude at the expense of the higher energy features for increasing FePc deposition temperature. Also the gaussian broadening increases (right axis). All these trends can be interpreted as due to a different degree of order of the molecular crystal (Fig. 1C), reflecting in different relative amplitudes of the observed split components and in an inhomogeneous gaussian braodening of the spectral lineshapes.
Changes in the IR intensity or a non-perfect temporal overlap of the IR and visible beams translate in a relative modulation of the resonant and non-resonant amplitudes (Supplementary Figure 6 and Supplementary Table 2).

Time-resolved spectroscopy of the electronic excited states. Supplementary Figure 7a shows a pseudo-color (intensity) representation of TR-2PPE as a function of the pump-probe delay. The spectra at each delay were normalized by subtracting the signal obtained at negative delay times, which is the background produced by the sum of 2PPE signals from both the pump and the probe taken separately. Such procedure isolates the pump-induced signal. We observed delay-dependent intensities in two kinetic energy regions of the plot, corresponding to short-lived (SL) and long-lived (LL) excited states. In order to improve the signal to noise ratio in our data we identify region SL, obtained by integrating the photoemission signal in a 0.3 eV wide kinetic energy region centered at 1.3 eV, and region LL, obtained in a 0.3 eV wide kinetic energy region centered at 0.6 eV. Supplementary Figure 7b shows the intensity profiles of SL and LL as a function of the pump-probe delay. Due to the fact that the full delay scan lasted for about 12 hours, a further normalization had to be performed for the longer delay part of the plot ($t > 1$ ps) in Supplementary Figure 7b, in order to remove intensity fluctuations due to the detection apparatus. This was achieved by dividing the intensity profile of LL by SL, since after the decay of the short-lived state there is no other time-dependent feature in that energy region. The short-lived state (SL) is fitted by a Gaussian curve yielding a full-width at half-maximum of 570 ± 100 fs, close to the cross-correlation of our pump-probe laser pulses. Therefore, we can conclude that the lifetime of the SL state is $\tau_{SL} < 570$ fs. The low energy long-lived state (LL) displays an exponential decay of the intensity, with the best fit yielding a time constant of $\tau_{LL} = 28 \pm 8$ ps.

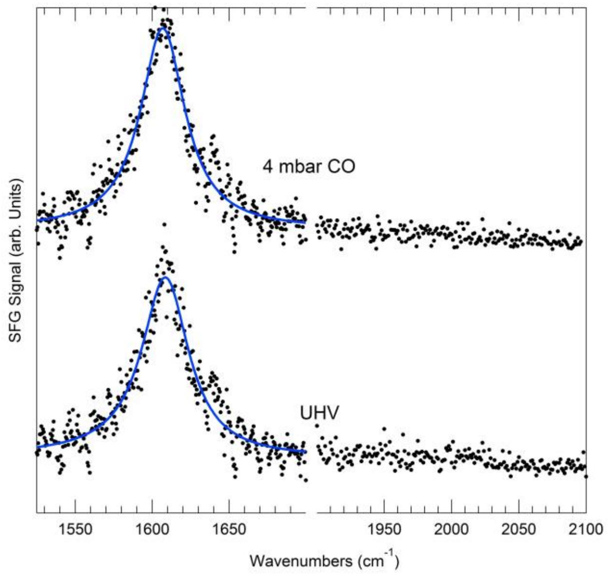

Supplementary Figure 1 IR-Vis SFG spectra of bare GR/Ir(111). Spectra were collected at room temperature at the graphene phonon (left) and C-O (right) stretching regions in vacuum (bottom) and at near-ambient CO pressure (top) [λ_{Vis} = 532 nm, ppp polarization].

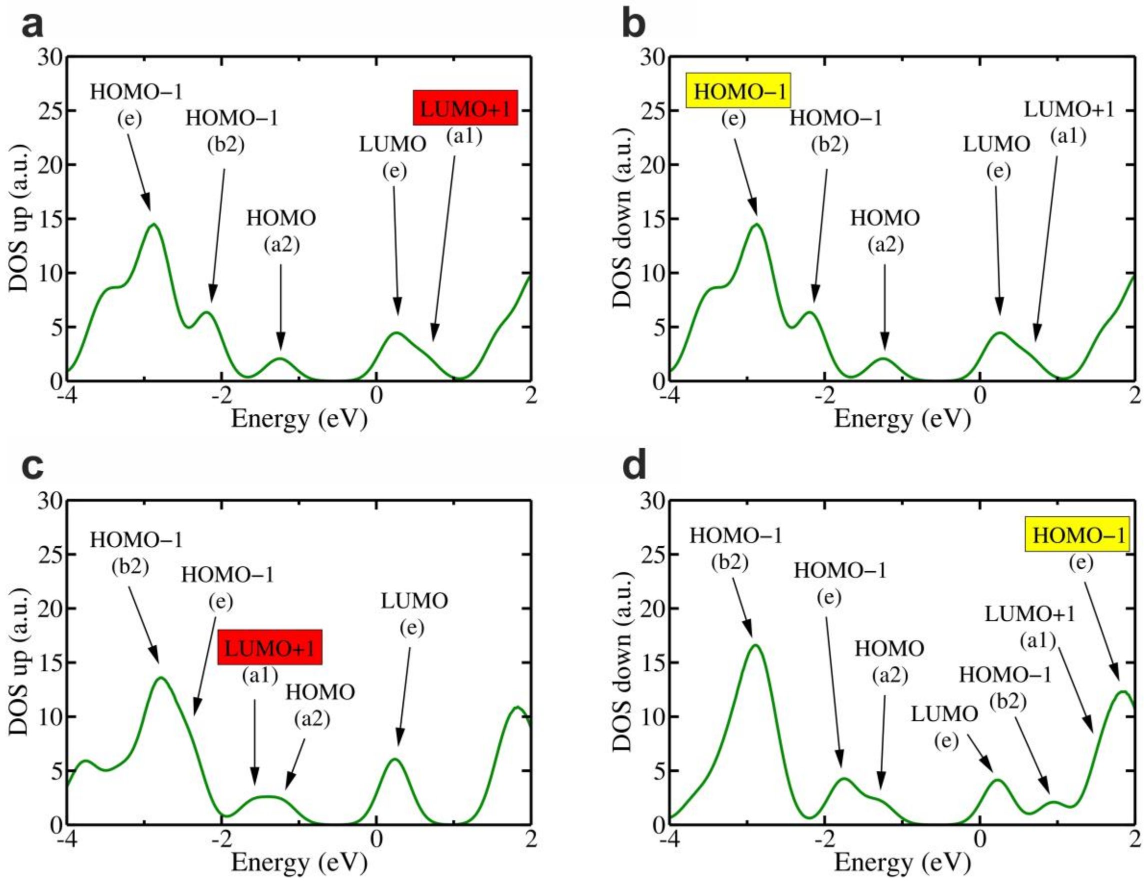

Supplementary Figure 2 Singlet density of states for: **a** spin up and **b** spin down electrons and triplet density of states for **c** spin up and **d** spin down electrons. The classification of the orbitals is made according to the irreducible representation of the C_{4v} group (which is the symmetry group of the isolated (CO)FePc molecule). The main differences between the singlet and the triplet states are highlighted in color, showing the emptying of a HOMO-1 state of the minority spins (yellow), and the filling of a LUMO+1 state of the majority spins (red). The Fermi level corresponds to the zero of the energy scale.

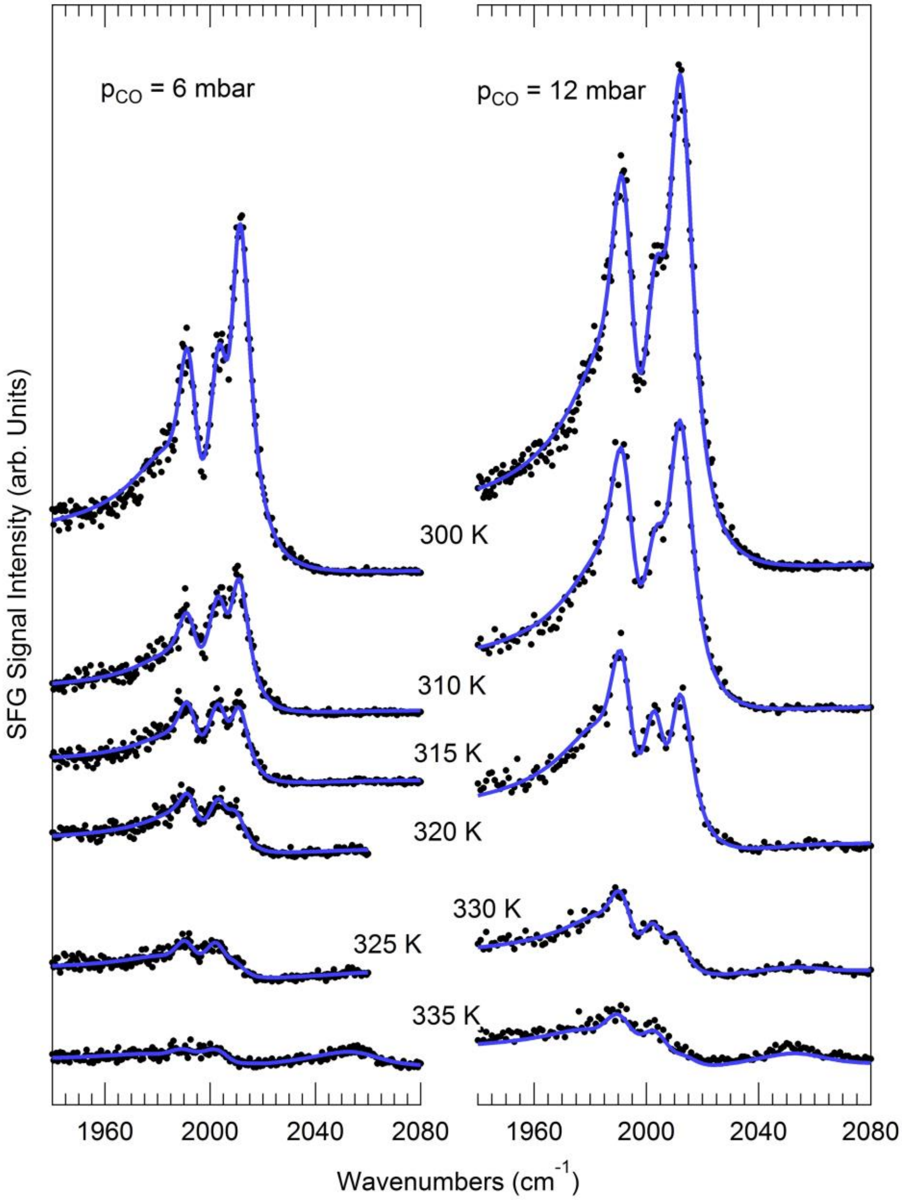

Supplementary Figure 3 Temperature evolution of the SFG signal. IR-Vis SFG spectra in the C-O stretching region of the carbonylated FePc monolayer on graphene as a function of temperature at near-ambient pressure in 6 (left) and 12 (right) mbar CO.

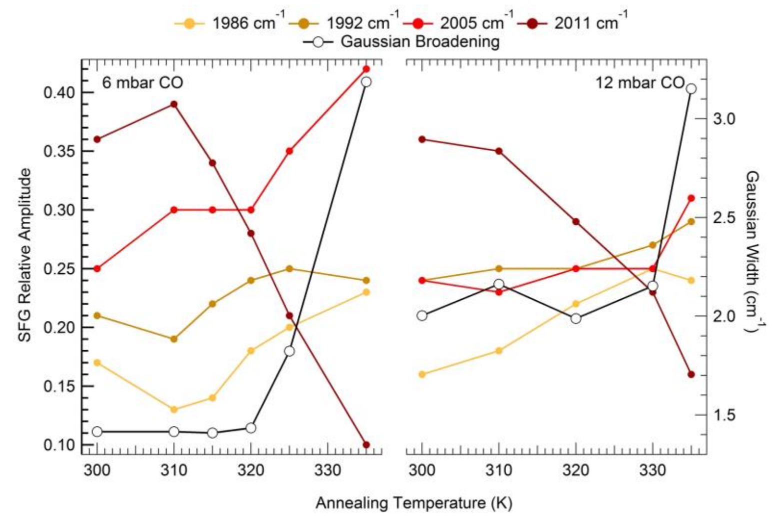

Supplementary Figure 4 Temperature evolution of the resonant amplitude and width of the C-O stretching features. SFG resonant signal relative amplitude (left scale) and Gaussian width (right scale) as a function of the annealing temperature for the carbonylated FePc monolayer at 6 (left) and 12 (right) mbar CO; data are obtained by fitting the spectra reported in Supplementary Figure 3.

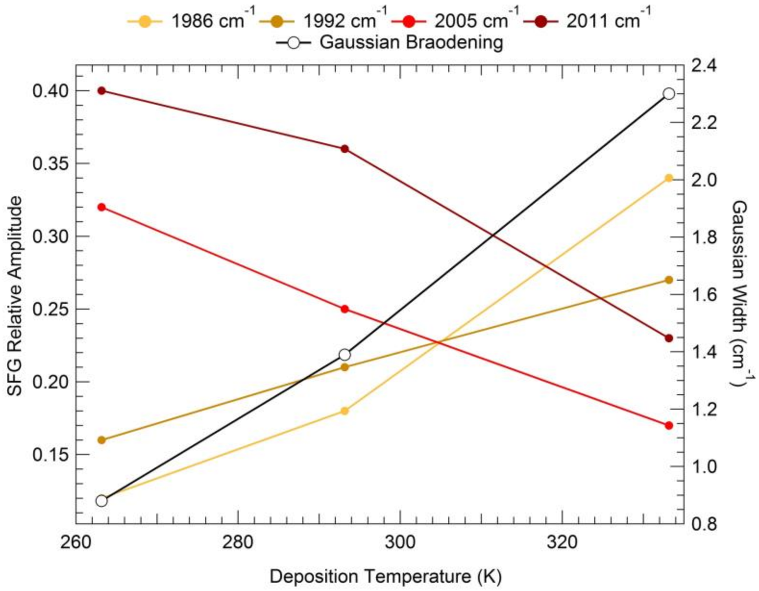

Supplementary Figure 5 Resonant amplitude and width of the C-O stretching features as a function of the layer pre-annealing, which reflects in a different degree of order of the 2D crystal. SFG resonant signal relative amplitude (left scale) and Gaussian width (right scale) for the carbonylated FePc monolayer in 10 mbar CO measured at room temperature.

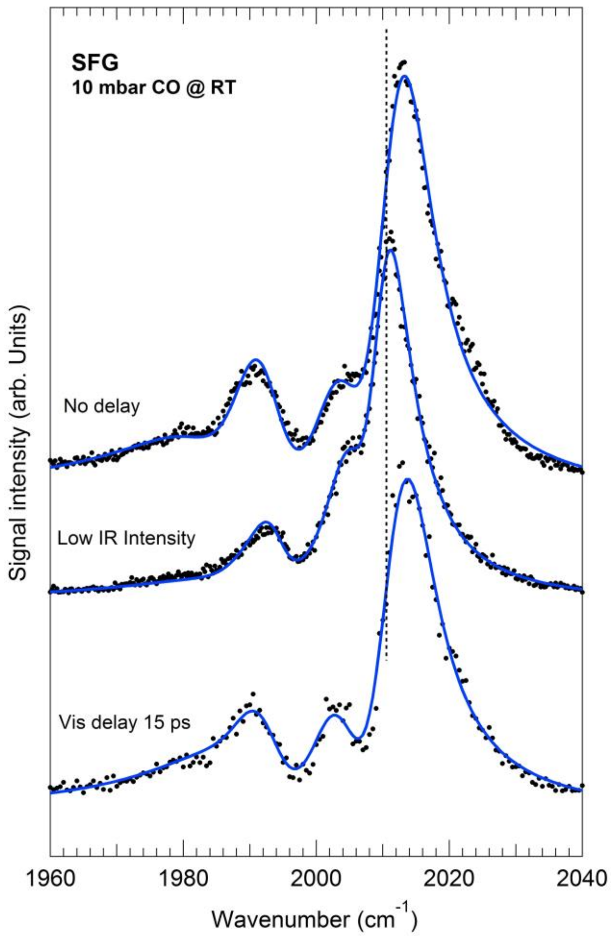

Supplementary Figure 6 IR-Vis SFG spectra in the C-O stretching region as a function of the laser beam parameters. Evolution of the SFG signal form the carbonylated FePc monolayer on graphene at room temperature in 10 mbar CO under different illumination conditions.

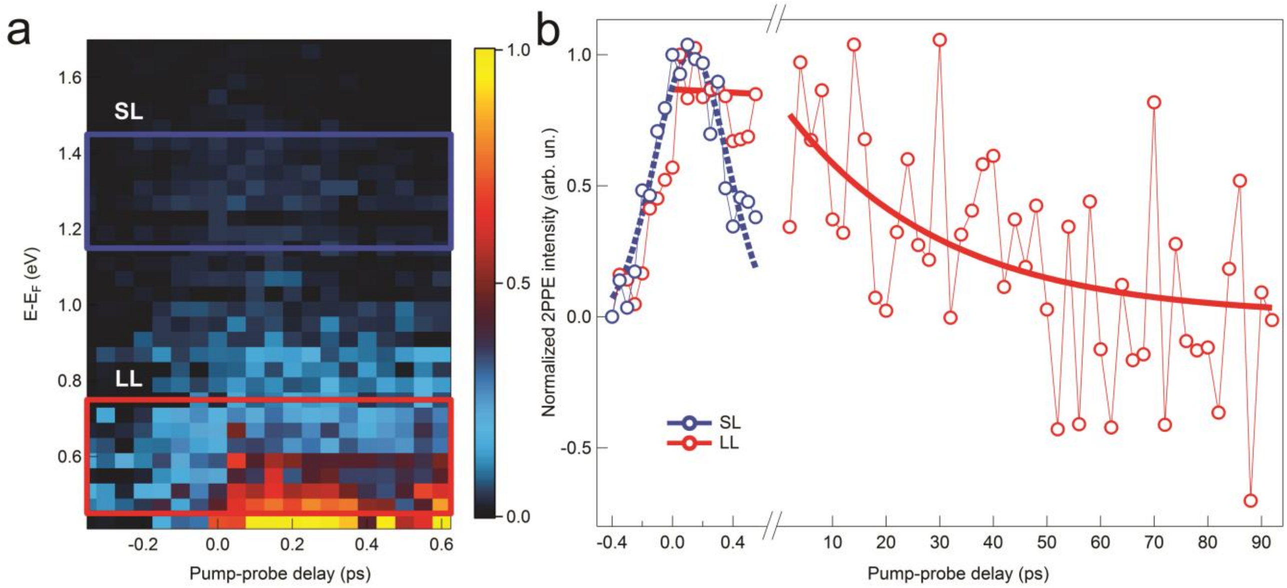

Supplementary Figure 7. (a) Pseudo-color plot of the TR-2PPE spectra for the FePc monolayer on Gr/Ir(111). The color scale represents the normalized photoemission intensity. The spectra were recorded with $h\nu_1 = 2.4$ eV (pump) and $h\nu_2 = 4.8$ eV (probe). The integrated intensities for the two regions delimited by the blue (SL) and red (LL) contours are displayed in (b). SL (blue markers) has been fitted with a Gaussian peak (blue, dashed line), while for LL (red markers) an exponential decay (red, solid line) has been adopted.

Parameter	Reference			
	Fig. 3 (ordered layer)	Fig. 3 (disordered layer)	Fig. 4 (multilayer)	Fig. 4 (monolayer)
$A^{(NR)}$ (arb. units)	0.40	0.44	1.37	0.10
Gauss σ (cm^{-1})	0.8	2.3	0.0	0.1
$A_1^{(RES)}$ (arb. units)	2.2	8.2	-	4.1
$\Delta\varphi_1$ (°)	50	50	-	50
ω_1 (cm^{-1})	1986	1986	-	1985
Γ_1 (cm^{-1})	8	8	-	8
$A_2^{(RES)}$ (arb. units)	3.4	6.5	-	7.1
$\Delta\varphi_2$ (°)	333	333	-	333
ω_2 (cm^{-1})	1993	1991	-	1994
Γ_2 (cm^{-1})	4	4	-	4
$A_3^{(RES)}$ (arb. units)	7.1	4.0	-	10.6
$\Delta\varphi_3$ (°)	356	356	-	356
ω_3 (cm^{-1})	2005	2004	-	2005
Γ_3 (cm^{-1})	4	4	-	4
$A_4^{(RES)}$ (arb. units)	8.6	5.5	20.0	21.6
$\Delta\varphi_4$ (°)	300	300	176	300
ω_4 (cm^{-1})	2010	2011	2012	2010
Γ_4 (cm^{-1})	4	4	5	4

Supplementary Table 1 Best fitting parameters of the IR-Vis SFG spectra reported in Figs. 3 and 4, according to the effective susceptibility as described above.

Parameter	Reference		
	High IR Intensity	Low IR Intensity	Vis delay 15 ps
$A^{(NR)}$ (arb. units)	0.08	0.10	0.03
Gauss σ (cm^{-1})	1.8	0.1	1.8
$A_1^{(RES)}$ (arb. units)	8.5	4.1	3.0
$\Delta\varphi_1$ (°)	50	50	50
ω_1 (cm^{-1})	1985	1985	1988
Γ_1 (cm^{-1})	8	8	8
$A_2^{(RES)}$ (arb. units)	8.5	7.1	2.8
$\Delta\varphi_2$ (°)	333	333	333
ω_2 (cm^{-1})	1992	1994	1992
Γ_2 (cm^{-1})	4	4	4
$A_3^{(RES)}$ (arb. units)	8.3	10.6	3.7
$\Delta\varphi_3$ (°)	356	356	356
ω_3 (cm^{-1})	2004	2005	2004
Γ_3 (cm^{-1})	4	4	4
$A_4^{(RES)}$ (arb. units)	15.9	21.6	6.5
$\Delta\varphi_4$ (°)	300	300	300
ω_4 (cm^{-1})	2011	2010	2012
Γ_4 (cm^{-1})	4	4	4

Supplementary Table 2 Best fitting parameters of the IR-Vis SFG spectra reported in Supplementary Figure 6, according to the effective susceptibility as described above.

Supplementary references

1. Scardamaglia, M. *et al.* Graphene-induced substrate decoupling and ideal doping of a self-assembled iron-phthalocyanine single layer. *J. Phys. Chem. C* **117,** 3019–3027 (2013).
2. Scardamaglia, M. *et al.* Metal-phthalocyanine array on the moiré pattern of a graphene sheet. *J. Nanoparticle Res.* **13,** 6013–6020 (2011).
3. Reuter, K. & Scheffler, M. Composition, structure, and stability of RuO2(110) as a function of oxygen pressure. *Phys. Rev. B* **65,** 035406 (2001).
4. Lovas, F. J. NIST Diatomic Spectral Database. Available at: http://www.nist.gov/pml/data/msd-di/index.cfm.
